# Unravelling the nuances: A scoping review on fatherhood and men’s participation in antenatal care in rural Sub-Saharan Africa

**DOI:** 10.1371/journal.pone.0332629

**Published:** 2025-09-17

**Authors:** Anthony Shuko Musiwa, Webster Mavhu, Owen Nyamwanza, Agatha Nyambi, Maya Stevens-Uninsky, Nadia Rehman, Naharin Sultana Anni, Roseline Dzekem Dine, Elizabeth Chadambuka, Rachel Couban, Lawrence Mbuagbaw

**Affiliations:** 1 Department of Health Research Methods, Evidence, and Impact, McMaster University, Hamilton, Ontario, Canada; 2 Centre for Research on Children and Families, McGill University, Montreal, Quebec, Canada; 3 Department of International Public Health, Liverpool School of Tropical Medicine, Liverpool, United Kingdom; 4 Centre for Sexual Health & HIV/AIDS Research Zimbabwe, Harare, Zimbabwe; 5 Department of Global Health, McMaster University, Hamilton, Ontario, Canada; 6 Department of Community Engagement and Social Science, Rinda Ubuzima, Rwanda; 7 Department of Health Sciences, Africa University, Mutare, Zimbabwe; 8 Faculty of Health Sciences, McMaster University, Hamilton, Ontario, Canada; 9 Department of Anesthesia, McMaster University, Hamilton, Ontario, Canada; 10 Department of Pediatrics, McMaster University, Hamilton, Ontario, Canada; 11 Biostatistics Unit, Father Sean O’Sullivan Research Centre, St Joseph’s Healthcare, Hamilton, Ontario, Canada; 12 Centre for Development of Best Practices in Health, Yaoundé Central Hospital, Yaoundé, Cameroon; 13 Division of Epidemiology and Biostatistics, Department of Global Health, Stellenbosch University, Cape Town, South Africa; Caleb University, NIGERIA

## Abstract

**Introduction:**

Men’s participation in antenatal care (ANC) in sub-Saharan Africa (SSA) is shaped by diverse conceptions and experiences of fatherhood. However, most discussions rely on biomedical models that typically view men’s participation narrowly as a strategy to increase ANC uptake in mainstream health facilities, often marginalizing culturally specific forms of participation. We aimed to consolidate the existing literature on the complex nuances of how attitudes, knowledge, variations in involvement, and decision-making dynamics influence men’s participation in ANC in rural SSA.

**Methods:**

Following the scoping review methodology developed by Arksey and O’Malley, we searched ten databases (African Index Medicus, Africa Journals Online, CINAHL, Cochrane Library, EMBASE, MEDLINE/PubMed, PsycINFO, Sociology Collection, Social Sciences Abstract, Social Sciences Citation Index) for peer-reviewed articles published between January 1st, 2000, and October 31st, 2024. We included only studies that systematically analyzed primary or secondary data to examine fatherhood and men’s participation in ANC in a rural setting in SSA. We applied no language restrictions.

**Results:**

We identified 7665 articles, full-text reviewed 797 articles, and included 77 articles that reported 58 qualitative, 6 quantitative, and 13 mixed-methods studies conducted in 15 SSA countries. We identified nine themes under three categories addressing our review’s objective: 1) three themes described men’s attitudes and knowledge around participating in ANC; 2) four themes depicted variations in men’s participation in ANC throughout pregnancy; and 3) two themes described how men’s participation in ANC was shaped by largely collaborative communal decision-making structures in rural SSA.

**Conclusion:**

While heterogeneous, the existing body of evidence highlights contextually-valid and socioculturally meaningful nuances that reflect the lived realities of fatherhood and men’s participation in ANC across rural SSA. Policymakers and practitioners should leverage these nuances as strengths, and further research should employ Afrocentric approaches to better understand these issues.

## Introduction

Despite significant progress over the years, sub-Saharan Africa (SSA) experiences the worst maternal and child health outcomes among all world regions [1,2]. A woman in SSA is 89 times more likely to die from pregnancy-related causes than a woman in Western Europe, the region with the best outcomes [2]. A child in SSA is 16 times as likely to die before their first birthday compared to a child in Western Europe [1]. The leading direct causes of maternal deaths in this region include obstetric hemorrhage, hypertensive disorders, infections, and non-obstetric complications, while for infants, the causes include perinatal asphyxia, low birthweight, and infections [3,4]. Poor social determinants, such as low income, limited education, unemployment, and lack of healthcare access, also contribute to high maternal and infant mortality in SSA [3,5]. As such, many SSA countries have implemented policies to enhance the participation of men, fathers, husbands, or male partners (hereafter, *men’s participation*) in antenatal care (ANC) [6,7]. Men’s participation means “the involvement (…), engagement or support of men in all activities related to maternal [and child] health” [8] (p1). ANC is the “care” provided to expectant women and their families by “skilled providers” to ensure the best health outcomes (we put “care” and “skilled providers” in quotations to signify that their meanings vary by context) [9,10].

In many SSA countries, involving men is largely used as a strategy to improve the use of all maternal and child health services especially within formal healthcare systems [6,11]. This article focuses on ANC because it is more consistent and has a greater impact on maternal and child health outcomes, including safer deliveries [12,13]. In contrast, childbirth care is more unpredictable, and postpartum care may come too late to prevent avoidable complications [12,13]. Despite mixed evidence about its impact on ANC use [14,15], men’s participation has been associated with many other health benefits for mothers and children, including significant reductions in pregnancy-related deaths [16–19]. However, current reviews indicate that men’s participation in ANC and maternal and child health remains low across SSA [17,20], pointing to a need for more in-depth research to inform relevant responses [7,21].

Men’s participation in ANC and maternal and child health in SSA is shaped by location-specific experiences of fatherhood [22,23]. Across this region, men or fathers are typically expected or required to be family leaders, providers, protectors, and nurturers [24,25]. Their efforts complement those of other physical and spiritual relational actors (women, children, ancestors, God, the environment, animals) in building thriving families and communities [26,27]. This complementarity reflects the communal and interdependent ways of being and living characteristic across SSA [27,28]. In this context, community means relating with others in harmony through sharing a sense of identity and acting in solidarity with other physical and spiritual actors [28,29]. In ANC, pregnancy, and childbirth situations, people—including men—in SSA collaborate with each other, often drawing ANC from local African indigenous and biomedical care systems concurrently, to ensure the best possible outcomes [9,30,31]. In our related work [32], we documented such relational contexts and men’s specific responsibilities within those contexts. Although ANC pluralism—that is, the practice of drawing on diverse types of care to support health and well-being during pregnancy and childbirth [30]—and familial and communal collaborations are widespread and often yield positive outcomes in many African societies, they remain underexplored in conventional analyses of men’s participation in ANC. Their complex nuances have not been systematically reviewed in the existing literature.

Instead, current reviews typically use (originally Western) biomedical definitions that focus on men’s direct involvement (or lack thereof) in health facility-based ANC programs, mainly male companionship at ANC visits or birth, birth preparedness, complication readiness, and involvement in antenatal education sessions [8,33,34]. These reductionist [33] measures are often promoted as the “correct” behaviors for men in ANC [31,32]. Men who do not practise those behaviors are disparaged as endangering [3] or displaying “a general neglect or disregard” [34](pS100) towards women’s and children’s health and wellbeing due to “inadequate knowledge, feigned unavailability and ignorance” [35] (p5). Forms of ANC and men’s participation grounded in biomedical approaches, such as those established by the WHO, are narrowly centered on individualized interventions [10,11]. Echoing a point we made above, these approaches systematically neglect African indigenous forms of ANC and men’s participation rooted in an African relational health model [31,36].

Furthermore, men in SSA are also normally portrayed as controlling or dominant in decision-making around ANC or other similar issues [37,38]. However, many across the region demonstrate commitment and contribute to ANC through performing specific responsibilities that are expected or required of them within their local sociocultural contexts [22,39]. To some degree, they know about ANC, pregnancy, and childbirth, drawing from their local cultural and biomedical knowledge systems and experiences [40–42]. And decision-making structures in SSA are certainly much more communal than male dominated, and shape men’s participation in ANC in many ways [23,43].

Accordingly, we conducted this review aiming to consolidate the existing scientific literature on the complex nuances of how attitudes, knowledge, variations in involvement, and decision-making dynamics shape men’s participation in ANC in rural SSA. While men’s participation in maternal and child health has been widely documented, most studies conceptualize participation in instrumental terms (e.g., practical support, clinic attendance, financial provision). Our review adds novelty by using context-specific, relational perspectives to explore how fatherhood is co-constructed through everyday caregiving, extended family interactions, and pluralistic care navigation in rural SSA—dimensions often overlooked in biomedical framings. We focused on rural SSA due to its distinct dynamics relative to urban SSA [44]. We are not aware of any reviews that have examined fatherhood and men’s ANC participation through the lens of relational care, communal ethics, and ANC pluralism in rural SSA.

While our findings can inform efforts to rethink men’s participation in ways that bridge biomedical and non-biomedical practices, our main goal was to deepen understanding of the nuanced forms of fatherhood and men’s participation within culturally pluralistic and community-centered societies across SSA. We acknowledge that some global health approaches gesture toward integrating biomedical and non-biomedical forms of ANC [6,7]. However, many often retain biomedical logics of task-shifting or outreach. In contrast, this review centres local epistemologies to explore how plural care systems already operate organically, with men and extended kin embedded in caregiving and decision-making roles often unacknowledged in formal health models. These findings can be used to inform the creation of culturally-appropriate responses and further research.

## Materials and methods

### Design

We chose the scoping review method because it is suitable for consolidating existing knowledge about an issue of interest based on carefully selected studies addressing that issue [45,46]. We adopted Arksey & O’Malley’s [46] seminal methodology, which has been improved over the years [45,47], including a more recent update by the Joanna Briggs Institute [48]. To guide our review, we developed a detailed protocol that has been published elsewhere [49]. We employed the Preferred Reporting Items for Systematic Reviews and Meta‐Analyses Extension for Scoping Reviews (PRISMA-ScR) guidelines in preparing this manuscript (see supplement S1 File) [50].

### Review questions

To achieve the aims of our scoping review, we addressed the following questions:

1) What are men’s attitudes and knowledge around participating in ANC in rural SSA?
2) How does men’s participation in ANC play out in rural SSA?
3) What decision-making structures shape men’s participation in ANC in rural SSA?

### Search strategy

With assistance from a university research methodologist, we drafted a robust search strategy to identify relevant scientific literature through ten electronic databases: African Index Medicus, Africa Journals Online, CINAHL, Cochrane Library, EMBASE, MEDLINE/PubMed, PsycINFO, Sociology Collection, Social Sciences Abstract, and Social Sciences Citation Index. Interested readers can access our search strategy attached to our protocol [49]. To conduct the search, we used key search terms and their variations, including “men”, “father”, “participation”, “involvement”, “prenatal”, and the names of all countries and regions in SSA. An initial search was conducted on February 28th, 2024, followed by an updated search on October 31st, 2024. To ensure a more thorough search, we also conducted reference checking of all included studies [51].

### Study selection

We selected studies relevant to our review through a combination of title/abstract screening and full-text review steps. To reduce bias and ensure a more rigorous review, we employed a complete dual review strategy whereby two reviewers separately screened the same articles. We resolved all conflicts through discussion and consensus. Our eligibility criteria were peer-reviewed articles reporting studies that (1) examined aspects of fatherhood and men’s participation in ANC, (2) were conducted in a rural setting(s) in SSA, (3) systematically analyzed primary or secondary data to draw data-driven inferences, (4) applied any design (quantitative, qualitative, mixed methods), and (5) were published between January 1st, 2000, and October 31st, 2024. We did not place any language restrictions. We defined “rural” as areas outside cities and towns, characterized by open spaces, low population density, and a slower pace of life [52]. We targeted articles that explicitly stated that the studies they reported were conducted in a rural area. Our baseline year aligned with the period when attention to men’s participation in maternal and child health started increasing in SSA and internationally, particularly after the 1994 International Conference on Population and Development in Cairo, Egypt [53,54]. We used a PRISMA flow-chart to track all articles included and excluded (and reasons for such) in the review, which helped in understanding any biases and implications pertaining to our findings [55].

To conduct title/abstract screening, ASM and LM independently test-ran our screening tool using 10 randomly-selected articles. The tool was revised based on this pilot test before additional reviewers (AN, MSU, NR, NSA, ON, RDD, WM) were invited to screen the rest of the articles, with ASM as the second reviewer for each additional reviewer. At the full-text review stage, ASM and LM separately test-ran our screening tool on 10 articles randomly selected from the articles included after title/abstract screening. The tool was revised after this test-run before AN, ASM, MSU, NR, NSA, ON, RDD, and WM full-text reviewed the remaining articles to select the final set of articles. Each reviewer independently screened a unique set of articles, with ASM working as the second reviewer. Our final screening tools are attached as supplement S2 File.

### Data extraction

The dataset for this review included key characteristics (names of authors, year of publication, country, study setting, study objective, research approach and design, data collection methods) and key findings extracted from each of the included articles. ASM and LM first piloted the tool used to extract these data on five randomly selected articles. After finalizing this tool (see supplement S2 File) based on this test run, five additional reviewers (AN, MSU, NR, NSA, RDD) extracted data from the remaining articles, with ASM and LM overseeing all extractions to ensure consistency and rigor.

### Data analysis

We analyzed the key study characteristics extracted from the included articles using descriptive statistics such as counts and presented them using tables and narratives. We thematically analyzed the key findings from the articles using QDA Miner Lite v3.0.6 software [56,57]. After thoroughly reading and understanding the extracted key findings, two reviewers (ASM, LM) intuitively coded and themed data from 15 articles randomly selected from the pool of included studies. This initial coding and theming facilitated the creation of an initial codebook that was used to guide additional reviewers (AN, MSU, NR, NSA, RDD) in analyzing the remaining data. We implemented iterative and reflexive methods [57] in coding and analyzing our data, including having regular team meetings and ongoing discussions. This approach facilitated the continuous development and refinement of our codebook and analysis through, for example, merging overlapping codes and themes and creating new ones where necessary. Finally, we combined all analyses to get the overall picture painted by our data and findings, including all consistencies, differences, and gaps [58,59]. All authors contributed to the development and refinement of this final analysis. No quality assessment of the included studies was conducted as this is not necessarily required of scoping reviews [45,47].

## Results

### Search outcomes

**Fig 1:**
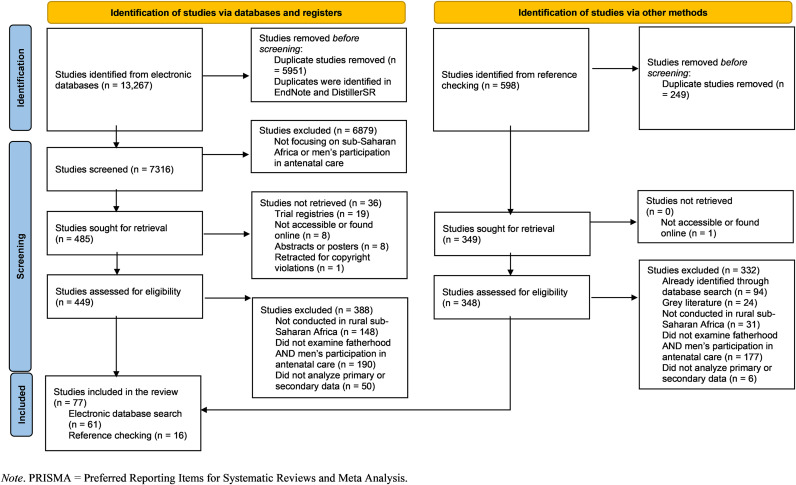
PRISMA flowchart depicting search outcomes.

We identified 13,865 articles from all searches. After importing the identified articles into an EndNote 21.4 reference library [60] and conducting an initial screening, we removed 6200 duplicates. Thereafter, we moved the remaining 7665 unique articles to a DistillerSR library [61] for the remainder of the review steps. We excluded 6916 articles at the title/abstract screening stage, leaving 797 articles for full-text review. After a full-text review, we retained 77 articles for inclusion in this review. More detailed outcomes of our search and inclusion/exclusion criteria are presented in Fig 1.

### Description of included studies

The 77 articles included in this review reported studies conducted in 15 countries across SSA and published from January 1st, 2005, to October 31st, 2024 (dates inclusive). All articles except one [62] were written in English. Based on the African Union’s categorization of African regions, Eastern Africa had the highest number of articles (*n* = 40) while both Central and Northern Africa had none. By country, most articles came from Tanzania (*n* = 17), followed by Ghana (*n* = 13) and Kenya (*n* = 11). Most studies were qualitative and conducted exclusively in community settings. The most common data collection methods were focus groups discussions and in-depth interviews. Participants’ ages across all studies ranged from 15 to 83 years for males and 15–76 years for females. A summary of the characteristics of the included studies is provided in Table 1 while a more detailed presentation is in supplement S3 File.

**Table 1:** Summary of the key characteristics of the included studies.

Characteristic	Number of studies
**Region** ^**#**^	
Eastern Africa	40
Western Africa	26
Northern Africa	0
Southern Africa	14
Central Africa	0
**Setting**	
Community (e.g., village)	35
Health facility (e.g., clinic)	15
Both community and health facility	27
**Design**	
Qualitative	58
Quantitative	6
Mixed methods	13
**Approach**	
Case study	47
Cross-sectional study	17
Other	13
**Data collection method** ^**#**^	
Focus group discussions	55
In-depth interviews	52
Survey questionnaire	19

*Note*: ^#^ = total number does not add up to the number of *included* studies because some studies covered multiple countries or implemented several methods.

### Review findings

We identified nine themes under three categories. The first category has three themes depicting men’s attitudes and knowledge around participating in ANC. The second has four themes describing variations in men’s participation in ANC throughout pregnancy. The last group comprises two themes that describe decision-making structures that shape men’s participation in ANC. Together, these nine themes highlight complex nuances whereby men participate in ANC, pregnancy, or childbirth within spaces carved out for them according to context-specific sociocultural conceptions and experiences of fatherhood in rural SSA. We present the themes identified from each included article in a table in supplement S4 File (tab 3 labelled *S*ummary Themes**), and a synthesis of our findings in the following sections.

#### A. Men’s attitudes and knowledge around participating in ANC.

1) **Men shared a sense of responsibility to participate in ANC, pregnancy, or childbirth**

In our previous work [32], we synthesized current knowledge around the perceived or enacted responsibilities of men in ANC in rural settings across SSA. We reported that men were expected or required to be family leaders, decision-makers, providers, protectors, advocates, advisors, nurturers, and helpers. In this section, we build on these prior findings by highlighting men’s salient motivations for perceiving or enacting those responsibilities in ANC situations in rural SSA. From our review, 32 studies conducted in Ethiopia [63], Ghana [40,62,64–67], Kenya [68–71], Malawi [41,72,73], Mozambique [74], Nigeria [75,76], Rwanda [77], Sierra Leone [78,79], South Africa [22,80], Tanzania [81–86], Uganda [87–90], and Zambia [68] documented that men were generally motivated to participate in ANC, pregnancy, or childbirth by a shared sense of responsibility as men, fathers, husbands, or spouses. While ANC served as the primary context for our analysis, this shared sense of responsibility extended beyond ANC alone. This broader engagement aligns with our review’s conceptual framing, which situates men’s participation within evolving notions of fatherhood, thereby encompassing the wider continuum of pregnancy and childbirth care across different systems of care.

According to our findings, men perceived that, by virtue of marriage or fatherhood, they had made a commitment to, or assumed the duty or obligation to, support their spouses and families in all matters, including ANC [40,62,67,68,76,87]. Some viewed this responsibility from a religious lens, arguing that their Christian values required them to support their spouses and families whether in or outside pregnancy situations [66]. Others opined that women (and children) were particularly susceptible to physical illness or spiritual attacks during pregnancy, and that it was their duty to protect them from all kinds of harm or injury [62].

Many perceived that ANC, pregnancy, or childbirth were financially demanding experiences and so they must work to provide the resources needed to meet those demands [65,82,89]. They noted that providing for their spouses and families took precedence over other forms of participation, including escorting their spouses to clinics [65,68,74]. However, one study found that in times of prolonged pregnancy-related illness, some men felt obligated to forgo work in order to look after their spouses, sometimes to the financial detriment of their families [81]. When men did not have adequate resources, they felt it was their responsibility to enlist support from relatives, friends, workmates, or others in their networks [81]. Those who partook in tasks traditionally viewed as women’s work (e.g., household chores, childcare) understood it was important to contribute a bit more in that area to help their spouses navigate the difficult times of pregnancy or childbirth [62,64,69,77]. As one study put it, “the men indicated that they cannot bear the pain their partners will go through”, demonstrating the empathy and care these men had for their spouses [62] (p8). Some men opined that men’s contributions towards domestic work should not just occur during pregnancy or soon after birth but be a permanent aspect of men’s roles [80].

Men who accompanied their spouses to ANC visits or who were present during childbirth—often in the face of cultural or social censorship over such actions—perceived their actions to be “the right thing” to do as husbands or fathers [73] (p9). They felt obligated to provide such companionship to ensure their spouses received appropriate care [71,74,78,83,86] or were treated well by care providers [71,84], or as a way of sharing in the challenges of pregnancy or childbirth or demonstrating solidarity, love, or care for their spouses [72]. The responsibility to support their spouses throughout pregnancy and to make the necessary preparations for the birth of the child motivated men to look for information or advice from care providers or others in their social networks [41,71,75,88,90]. Men’s responsibilities in ANC were also depicted in terms of the blame men received for failing to perform their duties as men, fathers, husbands, or spouses [22,67,70]. This included situations when (expectant) women made decisions and something went wrong [67]. Hence, men believed it was their duty to lead in all ANC, pregnancy, or childbirth matters to ensure everything would go well [67]. Ultimately, many drew satisfaction or pride from taking care of or supporting their spouses and families during the challenging times of pregnancy and childbirth [75].

2) **Men tended to know more about aspects of ANC that directly impinged on their responsibilities, drawing from local cultural and biomedical knowledge systems.**

Thirty-one studies conducted in The Gambia [91], Ghana [40,62,64,66,92,93], Kenya [35,68,71,94,95], Malawi [41,96], Nigeria [75,76,97–100], Tanzania [34,81,82,85,101–103], Uganda [88–90,104], and Zambia [68] depicted men as knowledgeable or aware of ANC, pregnancy, or childbirth aspects that impinged on their culturally-defined responsibilities. Men were able to identify physiological challenges [40,76,92,100], danger signs [41,71,76,97,100], risks [98], or causes of complications related to pregnancy or childbirth [41], with one study finding no misconceptions in men’s knowledge of these issues [41]. They demonstrated physiological and spiritual knowledge or awareness of pregnancy or birth-related complications [40,41,92], and perceived health facilities as best positioned to handle those complications and other issues [40,41,71,93,99]. Leaning more into their spiritual or traditional knowledge, some preferred to consult spiritual care providers to deal with problems they perceived as having spiritual causes, e.g., convulsions [41,92], or to have their spouses deliver at home supported by traditional midwives [41,104]. Men perceived their involvement in pregnancy, childbirth, or ANC as desirable or beneficial to ensure their spouses had healthy pregnancies and delivered safely [34,41,66,68,71,89,91,94,101–103].

Additionally, men knew that supporting their pregnant spouses during pregnancy was a critical aspect of supporting motherhood, ensuring their spouses would be effective in their roles as mothers [96,101]. Many were aware of their spouses’ ANC visit schedules [90] and understood it was important for their spouses to access ANC [34,41,68,89,94,97]. They understood that ANC was necessary to facilitate proper maternal and fetal health or development [33,40,83]. Some were willing to take a whole or part of the day off work to accompany their spouses to receive their ANC contacts [66,102,104], noting that “maternal health issues were to be treated with the seriousness they deserve” [66](p8). If/when they were unable to accompany their spouses to ANC clinics, men provided resources for the spouses to access care [68,75,104]. Men were aware that attending ANC visits with their spouses enabled them to access information that would help them support their spouses more effectively, including in terms of following guidelines provided by their care providers [41,68,75,81,82,88,94,102,104]. They knew that it was important to work hard to secure the resources needed to support their spouses and families and to prepare for the birth of the new child [66,71,81,82,85,92,101]. Men knew that it was their responsibility to avoid stress or risks of complications by reducing their expectant spouses’ workload around the house and ensuring they rested more [35,64,66,85,95,97,100]. They were aware as well that providing emotional support to their expectant spouses demonstrated togetherness, loyalty, or love during and after pregnancy [62,71,82,95] and helped reduce stress and potential complications [95].

3) **Men tended to not know more about the technical and minute details of ANC, especially in biomedical care contexts.**

Twenty-seven studies conducted in Ethiopia [63,105], The Gambia [106], Ghana [40,92,107], Kenya [35,108,109], Malawi [41,110], Nigeria [75,97,98,111,112], South Africa [22,113], Tanzania [34,81,102,114,115], and Uganda [87,88,90,116] documented that typically men were not well-versed with the more technical or minute details of ANC, pregnancy, or childbirth. According to this literature, men were ignorant or lacked experience in pregnancy or childbearing matters [40,75,81,97,111], and had limited or lower perceptions of the potential risks or complications associated with pregnancy or childbearing [40,41,92,98,106,107], including the relationships between convulsions (often attributed to spiritual causes) and eclampsia [41]. Men had poor or no knowledge of the range of ANC services provided at health facilities [34,35,108,115], did not see the value of knowing the kinds of ANC services offered at health facilities [34,105,111,115], lacked knowledge of what transpired at ANC clinics [113,114], did not understand the purpose, value, or potential benefits of ANC programs [63,88,109], lacked understanding of their roles in ANC besides participating in HIV testing and counselling and antenatal education sessions [110,112,114,116], and some were not aware of their spouses’ ANC appointment schedules [90] or why they should escort their spouses to their ANC visits [34,87,102]. Studies connected all these limitations to men’s limited or lack of involvement in ANC programs [110,115], limited or no support for their spouses’ engagement in ANC programs [35,109], limited or lack of support with household chores [35,106], poor health-seeking behaviors [22], and poor decision-making capacities around ANC, pregnancy, or childbirth issues [81].

#### B. Variations in men’s participation in ANC.

4) **Men’s participation in ANC varied based on the stage of pregnancy**

Thirty-three studies from Ghana [62,65–67,117], Kenya [68,70,71,95], Malawi [41,72,110], Mozambique [118,119], Nigeria [75,97,100], Rwanda [77], Sierra Leone [78,79], Tanzania [81,82,84,85,101,102,115,120–122], Uganda [104,116], Zambia [68], and Zimbabwe [122] demonstrated that men’s participation in ANC varied at different points throughout pregnancy. Many who perceived pregnancy as a delicate condition supported their spouses to access ANC more routinely [75,85,100]. They accompanied or arranged for other female relatives to escort their spouses to access ANC [66,78,81,82,102], arranged transportation to the preferred care facilities [78,81,102], advocated for their spouses to receive appropriate care [71,82], advised or decided for their spouses to deliver in public health facilities, which they perceived to be safer [81,82], strove to create peaceful home environments [68,82], or took up more domestic or childcare work to assist their spouses during pregnancy or after birth [68,71,75,77,82,102]. Additionally, men consistently participated through providing financial and material (e.g., food, clothing, shelter) support [65–68,70,72,75,78,79,81,82,84,85,97,101,102,110,116–120], protecting their spouses and families from all forms of harm or injury [62,65,75,82], and providing emotional support (love, care, intimacy, etc.) [65,75,78,81,82,101,122].

Other studies portrayed more episodic or intermittent participation. Men who did not perceive children as their concern until after they were born participated in child healthcare more than in maternal healthcare [116,118,119]. Yet, one study reporting how men actively supported—often for extended periods—their spouses in recovering from severe pre-eclampsia or eclampsia demonstrated that some men were in fact concerned with maternal health issues [81]. Men’s direct involvement in ANC programs was also described as less pronounced during the first trimester relative to later trimesters because expectant women often did not register with an ANC provider early into the pregnancy due to cultural norms around pregnancy disclosure [71,114,117]. Men who accompanied their pregnant spouses to health facility-based ANC programs normally did so on the very first visit, and fewer came for subsequent appointments [102]. Typically, the first ANC visit in these programs was mandatory for both spouses [84,102], though some men attended that first visit to make sure their spouses were well-received by care providers [82,84]. Men whose participation was intermittent appeared to be less involved in domestic work as well [101,102,121].

Furthermore, the literature demonstrated that men more actively advocated for their pregnant spouses to receive appropriate care during delivery compared to when their spouses attended routine ANC visits during pregnancy [82,84]. However, culturally, men were not allowed or were less willing to be physically present in or near birthing rooms when their spouses were giving birth [41,66,72,82,95,104,110]. As such, studies differed in their portrayals of men’s presence during childbirth. Some studies observed that some men were present in or near the delivery rooms when their spouses gave birth at home or at facilities ran by local traditional midwives compared to public health facilities [71,95,104]. Others reported that only a few men were actually present in or near birthing rooms whether at home, at the traditional midwives, or in health facilities [41,66,71].

Four studies reported that men participated or were more willing to participate in emergency situations, such as when there were pregnancy or birth-related complications or when their spouses were seriously ill [70, 85, 102, 118], as they perceived ANC as specialized care [85]. In such situations, men decided which forms of care to access and/or where to access them [65,70,97,118], provided the financial or material resources needed to access the selected care [65,102], or accompanied or arranged the transportation to take their spouses to the selected care facilities [65,85,102]. Still, three studies indicated minimal participation in emergency situations when the father was not physically present or had limited resources [67,79,97]. In such situations, the expectant woman, other senior relatives, or care providers made the necessary arrangements for the woman to access care [67,79,97].

5) **Men’s participation varied based on specific components of ANC**

Nineteen studies in The Gambia [91], Ghana [65,66,93,123], Kenya [70,71], Malawi [41], Nigeria [75,124], Sierra Leone [78,79], South Africa [113], Tanzania [81,82,84,102], and Uganda [125,126] indicated variations in men participation based on specific components of ANC. According to this literature, none, some or most (qualifier varied between studies) men did or were willing to pay for ANC fees and transportation fares for their spouses to access antenatal or delivery care [78,81,102,124,125], provide basic necessities (food, clothing, medical supplies, etc.) for their spouses and children throughout pregnancy [71,78,102,125], make decisions about antenatal or delivery care seeking [65,70,81,82,113], consult spiritual care providers such as traditional healers or soothsayers [41,65,93], or seek information or advice from care providers [113]. Few men—especially younger or first-time fathers and men with higher formal education levels—escorted or were willing to escort their spouses to ANC visits [65,75,78,81,84,102,113,124–126], to be present during delivery [65,66,75,78,91,113,124,126], or to partake in ANC consultations or education sessions [113].

6) **Men’s participation varied based on specific types of ANC**

Thirteen studies from Burkina Faso [127], Ghana [42,65,66], Kenya [71,95,109,112], Malawi [41], Nigeria [97], Tanzania [102,128], and Uganda [104] highlighted variations in how men participated in ANC based on specific forms of ANC, leveraging local African indigenous and biomedical ANC and knowledge systems. Some men supported their expectant spouses to access care from traditional midwives or traditional healers more than they did the ANC offered in health facilities [71,104,109]. Such men trusted or shared close relationships with traditional midwives more than they did biomedical health professionals [104,112], especially since traditional midwives often mediated domestic conflicts and supported men, their spouses and families to access both local African indigenous and biomedical forms of ANC [104]. In some studies, men preferred or were more willing to participate in traditional midwifery or traditional healing because of the lower financial costs, fewer medical supplies required, or close proximity of such forms of care relative to biomedical ANC [41,97].

Other studies documented that men preferred or more actively supported their pregnant spouses to give birth in health facilities than at home or assisted by traditional midwives, traditional healers, or other care providers [41,97]. Yet, as reported in a previous theme, the presence or absence of men in or near delivery rooms differed across studies. In emergency situations such as complications or serious illness, some men consulted traditional or spiritual care providers for complications (e.g., convulsions) they perceived to be caused by spiritual forces such as witchcraft, and engaged biomedical health professionals for conditions they did not suspect any spiritual foul play [41,97,127]. Others preferred to seek care from health facilities for all issues regardless of their perceived causes [41,97,127].

7) **There were tensions in men’s participation between different types of ANC**

Six studies from Ghana [40,92], Kenya [109], Malawi [41], Tanzania [129], and Uganda [104] highlighted tensions in conceptions or practices around men’s participation between different ANC systems. According to this literature, some men’s perceptions of risk in pregnancy matters were grounded in spirituality more than physiology [40,41,92]. Such men considered their engagement with the spiritual aspects of ANC, pregnancy, or childbirth as being more supportive towards their pregnant spouses than accompanying them to clinics [41,92]. Men’s preferences for traditional midwifery, traditional healing, or home birthing conflicted with public health recommendations to use biomedical forms of antenatal and delivery care, including HIV prevention programs [104,109]. Yet, in one study, most men preferred their spouses to utilize biomedical antenatal or delivery care, conflicting with local sociocultural norms around traditional midwifery or home birthing [41]. Finally, one study observed that gender-neutral biomedical notions of men’s participation diverged from the more gendered and culturally defined responsibilities of men (and women) in ANC matters such that “men are exposed to the contradictory and changing landscape of norms and expectations in relation to maternal health” [129](p106).

#### C. Decision-making structures that shaped men’s participation in ANC.

8) **Individualistic decision-making structures**
  (a) *The man expecting a child decided alone*

Thirty-three studies from Burkina Faso [127], The Gambia [106], Ghana [40,65,66,107,130], Kenya [69–71], Malawi [41,43,72,73], Mozambique [74], Nigeria [76,97–100], Tanzania [34,81–83,85,103,115,121,128,131], Uganda [104,126], and Sierra Leone [79] depicted that men exercised primary or sole decision-making authority around ANC and other household matters. This included making most if not all decisions about seeking antenatal or delivery care [43,65,71,72,74,76,79,83,97,98,115,126,128,130], choosing the care provider [65,71,81,97,98,104,128] and place for delivery [40,41,69–71,81,82,98], organizing transportation to the selected care facility [79,83], arranging which relatives (e.g., mothers- or sisters-in-law) would accompany the pregnant woman to ANC appointments or for delivery [40,66,128], choosing whether or not to pursue referrals to further care [127,128], ensuring all birth preparations were in place [83], distributing household resources towards ANC or other necessities [40,69,72,73,83,106,107], sharing domestic work [83,98,106], and seeking advice, information, or help from others where needed [65,71,72,83,128].

Four studies reported varying estimates of perceptions of men’s decision-making authority in ANC or related matters. One study in Malawi [41] found that 78% of men perceived that men decided on ANC seeking alone and nearly half (49%) perceived that men chose the place of delivery alone. About 62% of men and 59% of women in one study in Nigeria [97] expressed that men decided on the choice of ANC alone. In another study in Nigeria [98], 73% of participants stated that men decided on all household matters, including ANC. Several studies documented that expectant women needed permission from their spouses to engage with antenatal or delivery care [66,79,98,99,107,127,130]. Care providers had to discuss with expectant women about getting consent from their spouses before administering antenatal treatments like antiretroviral therapy for HIV [79,103], though most men in another study indicated their spouses did not need such consent [34]. In situations where they discussed ANC issues with their expectant spouses, or when both spouses initiated the need to access ANC, men exercised the final say and the responsibility to ensure that those decisions were carried out [71,83,100,131]. In emergency situations (e.g., pregnancy complications, serious illness), men decided the way forward, whether they were physically present or not [70,72,127,128].

(b) *The expectant woman decided alone*

Fourteen studies from Burkina Faso [127], Ghana [66,67,93], Malawi [41,43,72,73,110], Nigeria [97,99,100], Sierra Leone [79], and Uganda [89] documented perceptions or situations when expectant women exercised primary or sole decision-making authority on ANC or other issues in their households. One study enumerated that 9% of men indicated that women decided on ANC-seeking alone while 5% stated that men left the choice of place of delivery to their spouses [41], consistent with another study that demonstrated that women made decisions about accessing services such as ANC or child immunizations [100]. In yet another study, men preferred to leave all antenatal or delivery care decisions to their spouses [89]. Women also decided whether or not to invite their spouses to go with them for ANC appointments or to give birth [110]. After giving birth, they made the choice to either return to their matrimonial home or go and stay with their mothers or other senior female relatives (e.g., maternal grandmother) from their families who would care for and support them and their newborns as they recuperated [66]. Additionally, (expectant) women decided on or controlled food choices, procurement, preparation, or sharing in the household [73]. They exercised primary or sole decision-making authority on accessing care when they encountered emergencies (e.g., complications) [67,93], used their own resources, whether their spouses were present or not [127], were widowed or single/unmarried [127], or when their spouses worked far away [72].

9) **Collaborative decision-making structures**
  (a) *Both parents expecting a child decided together*

Thirteen studies conducted in Burkina Faso [127], Ethiopia [105], Kenya [94], Malawi [41,43], Mozambique [74], Nigeria [99,100], Tanzania [82,83,121], Uganda [89], and Rwanda [77] documented perceptions or situations when men and their spouses made ANC decisions together. In one of the studies, 10% of men stated that both spouses decided together on ANC seeking while 32% stated that spouses jointly chose their preferred place for giving birth [41]. Shared decision-making was described in the literature as occurring through open communication or discussion around ANC, household, financial, or related issues, and through integration of ideas from both spouses in making the necessary decisions [43,77,83,100]. Open communication or discussion entailed the spouses being free to approach or initiate a conversation about an issue, sitting down to discuss an issue, listening to each other’s opinions, and not hiding anything from one another [43,77,100]. Two studies found that (expectant) women did not necessarily need to seek permission but, instead, shared relevant information or discussed with their spouses on the best courses of action in navigating ANC [82,99]. Three other studies indicated that shared decision-making on ANC issues increased the likelihood of men being directly involved in ANC activities, taking more domestic or caregiving responsibilities, or making better financial decisions that benefitted their families [77,94,121].

(b) *Members of the extended family or local community decided*

Seven studies conducted in Burkina Faso [127], Ghana [42,67,130], Malawi [41,72], and Tanzania [128] demonstrated that extended family members—most commonly mothers, mothers-in-law, and grandmothers—had significant influence over decisions about ANC. In some cases, decisions were made by extended family or community actors in ways that reflect both influence and deference to local authority structures. Rather than formal consensus, these decisions reflected relational obligations and cultural expectations embedded in collective care. In one study in Malawi, 3% of men perceived that relatives decided on ANC seeking and 13% perceived that relatives chose the place of delivery on behalf of them and their pregnant spouses [41]. Senior female relatives, such as those listed above, exercised substantial influence over decisions around ANC seeking [41,72,127,130], choice of place of delivery [41,42], or completion of referrals for further care, including in emergency situations [127,128]. Those women were generally trusted for their deep knowledge or experiences around issues of ANC, pregnancy, and childbirth and for their critical roles in managing families or local communities, or maintaining social cohesion in terms of their traditions and norms [42,67,127]. Their dominance or authority significantly shaped the various forms or levels of men’s involvement in antenatal or delivery care spaces [67].

Four studies conducted in Burkina Faso [127], Ghana [93,130], and Tanzania [128] indicated that senior male relatives—typically the spouses’ fathers, fathers-in-law, grandfathers, and older brothers—exercised significant decision-making authority around ANC issues. Here again, some instances involved the relatives actually deciding on ANC, demonstrating their influence and respect for local authority as well as prevailing communal caregiving ethics. Pregnant women whose husbands were not physically present or those who were widowed or single needed the consent of a senior male relative—such as anyone listed above—to pursue activities outside the home, including accessing antenatal or delivery care [127,130]. Nonetheless, one study indicated they were free to seek care without such consent [93]. Any senior male relative(s) of the expectant woman had the authority to override any ANC-related decisions made by the woman or her spouse, particularly if the relative(s) disagreed with those decisions, based on their norms and values [127,128].

Nine studies from Ghana [42,65,93,117,130], Malawi [41,72], Nigeria [111], and Uganda [104] observed that local African indigenous care providers had significant influence over and, in some cases, made the decisions about ANC or related issues. In many SSA settings, traditional midwives and spiritual practitioners wield authoritative knowledge; however, their influence is relational and embedded in cultural trust networks, where guidance is enacted through social respect rather than coercive enforcement [9,36]. This reflects a different logic of authority than clinical hierarchy. Hence in one of the studies in this review, 13% of men perceived that men left decisions about the place for delivery to local traditional midwives [41], many of whom were mothers, mothers-in-law, or grandmothers of the men and women they served [42,104]. Traditional midwives leveraged their deep knowledge and experience to exert influence around access to specific forms of antenatal or delivery care [42,72,104]. Men’s preferences for the kinds of care provided by traditional midwives provided the midwives with more leeway to support those men and their spouses during pregnancy [42,72,104]. Yet, the midwives controlled which antenatal or delivery spaces men took part in [111]. Soothsayers provided families with spiritual guidance during pregnancy, enabling them to exercise some level of influence in the families’ antenatal or other decisions [65,93].

(c) *The parents expecting a child and members of their family and local community decided together*

Eight studies from Burkina Faso [127], Ghana [42,93,130], Malawi [43], Mozambique [74], Nigeria [76], and Tanzania [128] documented that ANC decisions were not dependent on one or a few parties. Instead, ANC, pregnancy, and childbirth were collective responsibilities involving the efforts of multiple parties from both spouses’ families and local communities [42,43,93]. ANC decision-making entailed multiple parties who made specific decisions on specific issues at specific junctures throughout pregnancy, based on their social positions or responsibilities in their families or local communities [42,76,127,128,130]. One study indicated that men were responsible for decisions about money and sexual relations, women dominated decisions about childbearing and domestic work, and both spouses jointly decided on pregnancy, contraception, food, child welfare, money, and other general issues [43]. Five studies observed that expectant women received information or guidance from care providers and shared with their spouses who, after reviewing such information or guidance either alone or jointly with the women, would consult other senior members of their families for their input [42,93,127,128,130]. Women also exercised discretion on what information they shared with their spouses, while both spouses often chose whether or not to consult other relatives [42,93,127,128,130]. While guidance provided by care providers may lack formal mechanisms for exploring alternatives, in pluralistic care contexts like SSA, decisions are often filtered through kinship, spiritual interpretation, and relational trust, making care pathways non-linear and socially negotiated [9,36].

## Discussion

This review synthesized current research around (some of) the complex nuances of fatherhood and men’s participation in ANC in rural SSA. We demonstrated that men’s orientations to participating in ANC were informed by their shared sense of responsibility as men, fathers, husbands, or male partners as well as varying gradations of knowledge or understandings of ANC, pregnancy, or childbirth. Men’s participation in ANC varied throughout pregnancy based on the stage of pregnancy, specific components of ANC, and different types of ANC. There were tensions in men’s participation between different types of ANC as well. Finally, men’s participation in ANC was shaped by decision-making structures ranging from individualistic (either the man or woman expecting a child decided alone) to more collaborative structures where both parents (shared) and multiple family and local community members (communal) decided together. We discuss these findings below.

The finding that men in rural settings across SSA are oriented to participate in ANC, pregnancy, or childbirth based on a shared sense of responsibility as men, fathers, husbands, or spouses is echoed in previous studies elsewhere in Guatemala [132] and Nepal [133]. As our prior work [32] and studies elsewhere outside SSA [134,135] have demonstrated, this responsibility manifests in men’s performance of specific responsibilities informed by prevailing norms and values in their sociocultural contexts. In addition, the finding that some men do, in fact, know about or understand ANC, pregnancy, or childbirth issues has been similarly documented in non-SSA studies in Bangladesh [136] and Brazil [137]. At the same time, the studies just cited above also demonstrated, like our review did, that other men do not know about or understand these issues. However, building on current studies, our review underlines that men in rural SSA generally tend to know more or understand better those aspects that directly impinge on their responsibilities in ANC matters. This finding points to a need to contextualize what counts as valid “knowledge” in empirical assessments of men’s knowledge to better understand their participation in ANC, pregnancy, or childbirth in rural SSA.

The finding that men’s participation in ANC varies at different points throughout pregnancy has been similarly documented in studies in The Cook Islands, Fiji, Papua New Guinea, Solomon Islands, and Vanuatu [138]. The variations in men’s participation based on specific antenatal or delivery care services in this review echo previous research in Colombia [139] and India [140]. Similar to our review, studies conducted in India [141], Mexico [142], and Pakistan [143] have documented variations and tensions in men’s participation between biomedical and non-biomedical ANC systems. All these findings point to complex nuances that characterize men’s participation in ANC. They demonstrate that men in rural parts of SSA typically participate in ANC at specific strategic junctures and spaces throughout pregnancy or childbirth, based on the norms and values set within their localities [66,78]. Additionally, our findings shine a light on the rarely-acknowledged but important ways men participate in ANC within local African indigenous systems such as traditional midwifery and spiritual care [92,104], forms of ANC as equally valid as biomedical ANC [30,144].

The finding that men in rural SSA have sole or primary decision-making authority on issues of ANC aligns with findings from previous research elsewhere in Pakistan [145] and Jordan and Saudi Arabia [146]. Similar to our review, studies conducted in Nepal [133] and Colombia [139] highlighted situations where expectant women made most or all decisions around ANC or other household matters. All these cited studies also documented shared decision-making norms and patterns between spouses on issues of ANC, similar to our review findings. Furthermore, our review finding of the significant decision-making role played by (extended) family or local community members in ANC matters, is similarly salient in previous research in China [147], Guatemala [148], and Nepal [149]. We connected our findings around the collaborative efforts of multiple family and local community members in deciding on aspects of ANC to similar literature in Iran [150] and Bangladesh, India, and Pakistan [151] as well. All these findings highlight complex ANC decision-making structures that implicate men and other relational actors, including women, in very nuanced ways across rural SSA. While network influences on maternal health behaviors are well-documented, our review contributes new insights into men’s embeddedness in caregiving and care-seeking as relational actors, not merely decision-makers.

Ultimately, our review contributes at least three critical insights to the existing literature. First, by highlighting men’s shared sense of responsibility and the specificities of their knowledge around issues of ANC, pregnancy, and childbirth, we shine a light on the contextually valid and socioculturally meaningful ways in which men approach and experience participation in ANC in rural SSA. We contrast these findings with conventional representations of men as disengaged from maternal and child health issues [34,35]. Such representations reflect policy and research gaps rather than lived realities, where many men contribute to ANC in ways not easily captured by biomedical metrics. Second, by depicting variations in men’s participation in ANC, we highlight the messy landscape of different forms and levels of participation at different junctures throughout pregnancy and across different ANC systems that complement and conflict with each other in rural SSA. We underscore the contextual determinacy of men’s participation in ANC that is often neglected by many biomedical approaches to this issue [33,78]. Finally, by describing the different decision-making structures that exist in rural SSA, we draw attention to the nuanced ways in which men collaborate with other relational parties on ANC issues in rural SSA. Even what we characterized as “individualistic” decision-making structures appear to be part of broader collective efforts involving multiple (extended) family and community members [42,43]. These findings reflect the communal ways of life and being that are characteristic in most settings across SSA [28,31].

## Implications for policy, practice, and further research

Our findings point to a need for ANC policies and practice that foster fatherhood and men’s participation in ways that reflect lived and sociocultural realities in rural SSA. Policymakers and practitioners must move away from (not beyond) deficit-based biomedical approaches that focus on men or local sociocultural factors as “risks” or “barriers”, to more holistic strengths-based Afrocentric models that recognize and embrace people (including men), cultures, language, and local resources and skills as critical assets in ANC and other maternal and child health issues [152,153]. This includes supporting the many positive contributions men make in ANC or related matters based on their cultural responsibilities by, for example, improving their capacity to provide through decent employment opportunities and lucrative subsistence agriculture and informal mining activities [24,39]. As well, we recommend reimagining “knowledge” in men’s participation issues by combining African indigenous knowledge and teachings around positive fatherhood [76,154] with the largely biomedical ANC education curricula prevalent in SSA. Collaborating with local traditional, religious, and spiritual leaders and other community stakeholders in these efforts is key [76,155].

We encourage policymakers and practitioners to acknowledge and embrace the complexity of men’s participation in ANC, meaning such participation does not necessarily have to occur in the same manner for all spouses, families, or communities. For example, as our review highlighted, direct involvement in ANC activities among some men (and even women) appears less pronounced in the first, relative to late, trimesters based on sociocultural norms around pregnancy disclosure [117]. In those situations, ANC providers can offer to engage spouses or families in ways that uphold their confidence until the parties are comfortable disclosing to “outsiders”. Traditional midwives have been shown to be effective in providing such discretionary care [30,104] and in ways that allow men to participate more actively [104]. Building on the last point, we urge policymakers and practitioners to embrace ANC pluralism [30] by working with local African indigenous care providers to enhance men’s participation in ANC. As our study revealed, some men feel more comfortable working with traditional midwives and spiritual care providers compared to biomedical care practitioners [41,92,104]. Collaboration across different care systems can foster men’s participation in ANC, including in biomedical care contexts.

Furthermore, we recommend policymakers and practitioners to employ family and community systems approaches in supporting families in making ANC decisions that center the health and wellbeing of women and children. We acknowledge and share concerns especially from public health professionals across SSA [83,127] and worldwide [156,157] around delays in care seeking that may be caused by complex decision-making norms. At the same time, we encourage more engagement and collaboration with families and local communities on the importance of timely ANC decision-making in ways that can still respect peoples’ cultures and ways of life. For example, families can be encouraged to make contingency decisions about ANC in advance to avoid family processes that may delay expectant women from accessing timely care. In addition, by characterizing men’s decision-making roles as hegemonic or dominating, much of the existing literature risks pitting men against women in ANC matters [7,37,38]. We encourage policymakers and practitioners to lean more into Afrocentric values that encourage family, community, responsibility, and complementarity between men, women, and other relational actors [25,26,158]. This does not mean an uncritical endorsement of norms or practices that are harmful to the health and wellbeing of women, children, and families.

Finally, this review indicates some critical future research pathways to inform efforts to foster positive fatherhood and men’s participation in ANC. First, more research employing various designs is required to examine how men make sense of their responsibilities in ANC and the kinds of knowledge they have or leverage in fulfilling those duties. Such studies could follow the leads of recent studies examining Afrocentric conceptions of fatherhood preparedness [22], responsibilities and identity [159], and “ideal” fatherhood [154] as they apply in maternal and child health issues, moving beyond the narrow biomedical definitions of men’s participation. Second, we recommend more (qualitative) studies around the complex nuances of men’s participation at different junctures during pregnancy and between different ANC systems. Such studies could, for example, compare men’s participation in ANC between the first, second, and last trimesters, and between traditional midwifery and biomedical care systems, consistent with the ANC pluralism that exists across SSA [30,144]. Finally, we suggest more qualitative studies, such as Mbweza’s et al. [43] and Jansen’s [42], to explore the complex ANC decision-making structures that exist across SSA. We encourage all these studies to employ Afrocentric conceptual/theoretical approaches, such as Afro-communitarianism [28], African postcolonial approaches [153], and Afro-feminism [158], to generate more culturally-appropriate understandings and tailored interventions.

## Limitations

Our review must be interpreted against its limitations. By excluding gray literature and other non-peer-reviewed articles, we likely missed other insights into fatherhood and men’s participation in ANC. We also could not locate a very small number of articles online or in hard copy. Yet, we believe our dataset was sufficiently saturated such that those exclusions did not significantly bias our findings. During analysis, we were inspired by the heterogeneity of conceptions and experiences of, and the methods used to examine, fatherhood and men’s participation in ANC in different rural settings across SSA. However, in an attempt to accurately synthesize the findings, which we believe we did, we lost some of these nuances. We encourage readers to refer to the cited studies to engage with any of our findings in more detail. Finally, we performed no quality assessments for the studies included in this review. While this is not necessarily required of scoping reviews [45,47], it may be a potential limitation.

## Conclusion

This review consolidates the existing scientific literature on the complex nuances of fatherhood and men’s participation in ANC in rural parts of SSA. Our findings demonstrated that men shared a sense of responsibility and varying levels of knowledge around ANC, pregnancy, and childbirth drawing from local cultural and biomedical knowledge systems. These attitudes and knowledge shaped their participation in ANC in different ways. Additionally, men’s participation varied based on different junctures throughout pregnancy, specific components of ANC, and different types of ANC, and there were some tensions in men’s participation between different ANC systems. Finally, we found different decision-making structures that shaped men’s participation, ranging from individualistic (either man or woman decided alone) to collaborative structures where both parents (shared) and multiple family and community members (communal) decided together. We encourage policymakers, practitioners, and researchers to use these findings to develop culturally-appropriate responses and further research to enhance fatherhood and men’s participation in ANC in (rural) SSA.

## Supporting information

S1 File Preferred Reporting Items for Systematic reviews and Meta-Analyses extension for Scoping Reviews (PRISMA-ScR) Checklist. (DOC)

S2 File Screening and data extraction tools. (DOC)

S3 File Characteristics of included studies. (DOC)

S4 File Identified studies and findings. (XLSX)
