# Supplementary material for: Unravelling the nuances: A scoping review on fatherhood and men’s participation in antenatal care in rural Sub-Saharan Africa
Source: PLoS One. 2025 Sep 17;20(9):e0332629. doi: 10.1371/journal.pone.0332629 (PMC12443264; doi:10.1371/journal.pone.0332629)
Supplement: S2 File — (DOC) [file pone.0332629.s002.doc]

# **S2_File: Screening and data extraction tools**

## **A. Title/abstract screening tool**

1. Does the title or abstract indicate that the study focused on men’s participation in antenatal care in sub-Saharan Africa?

Yes – Move to full-text screening

Maybe – Move to full-text screening

No – Exclude from further review

## **B. Full-text screening tool**

1. Was the study conducted in a rural area in sub-Saharan Africa?

Yes  No

1. Was the study focused on the roles of men or fathers during antenatal care?

Yes  No

1. Was the study focused on how men or fathers perceive fatherhood during antenatal care?

Yes  No

1. Was the study based on analysis of primary or secondary data (qualitative, quantitative, or mixed)?

Yes  No

1. Decision: Should this article be included in the review?

Yes - All four questions above answered “Yes”

No - At least one question above answered definitely “No”

## **C. Data extraction tool**

1. What is the article’s first author’s last name and year of publication?

_____________________________________________________

1. What language was the article written in?

English  French  Portuguese

Arabic  Other (specify)____________________________

1. In which country/countries was the study conducted?

*55 sub-Saharan countries were listed and assigned checkboxes, with instructions to check multiple countries where applicable. An “Other” (please specify)” section was provided for any sub-Saharan African country not listed*.

1. What was the primary aim(s) or objective(s) of the study?

________________________________________________________________________________________________________________________________________________

1. In what specific setting was the study conducted?

Community only  Health facility only

Both community and health facility

Other (specify)____________________________________

1. Did the study use any theoretical or conceptual framework?

Yes  No – Skip to Question 8!

1. What theoretical or conceptual framework(s) was used in this study?

_______________________________________________________________

1. What research approach was used in this study?

Qualitative  Quantitative

Mixed methods  Other (specify)________________________

1. What research design was used in this study?

Experimental – Go to Question 10!

Observational – Skip to Question 11!

1. What type of experimental research design was used in this study?

Randomized or quasi-randomized trial

Case-control study

Cohort study

Case report / case series

Other (specify)

1. What type of observational research design was used in this study?

Case study

Grounded theory

Cross-sectional study

Other (specify)

1. What specific population(s) was engaged in this study? *Check all that apply. For each population selected, state the number of participants numerically in the space provided*.

Fathers ___________

Male spouses____________

Other male extended family or local community members__________

Pregnant women__________

Mothers___________

Female spouses____________

Other female extended family or local community members __________

Biomedical health professionals (e.g., doctors, nurses-midwives) _______

Traditional care providers (e.g., traditional midwives) ______

Local community gatekeepers (e.g., traditional leaders) _________

Other (specify)______

1. What type of data was used in this study, irrespective of the research design employed?

Primary data only  Secondary data only

Both primary and secondary data

1. What method(s) was used to collect data in this study? *Check all that apply*.

Interviews  Focus group discussions  Survey / questionnaire

Observations  Other (specify)_________________________________

1. In which month/year did data collection for this study start? e.g., September 2021.

___________________________________

1. In which month/year did data collection for this study end? e.g., January 2022

___________________________________

1. Provide a summary of the main findings/themes of this study that specifically relate to why or how men or fathers experience specific roles in antenatal care?

________________________________________________________________________________________________________________________________________________________________________________________________________________________

1. Where participant quotes were provided in the article to support findings (see Findings section), choose one quote that best supports each finding/theme that you have extracted in the previous question. Also, indicate the page number(s) from where you extracted each quote.

________________________________________________________________________________________________________________________________________________________________________________________________________________________

1. Was funding information reported in this article?

Yes – Go to Question 20!  No – Skip to Question 21!

1. What was the source of funding for this study? Check all that apply.

Government  For-profit organization

Not-for-profit organization  Study did not receive any funding

1. If there is any information that has not been captured by the questions above which you think could help us address our scoping review question better, please provide it in the space below. If not, write “Not applicable”.

________________________________________________________________________________________________________________________________________________________________________________________________________________________
