# Supplementary material for: Unravelling the nuances: A scoping review on fatherhood and men’s participation in antenatal care in rural Sub-Saharan Africa
Source: PLoS One. 2025 Sep 17;20(9):e0332629. doi: 10.1371/journal.pone.0332629 (PMC12443264; doi:10.1371/journal.pone.0332629)
Supplement: S3 File — (DOC) [file pone.0332629.s003.doc]

# **S3_File: Characteristics of included studies.**

| **Authors and publication year*** | **Country and study setting** | **Primary objective** | **Approach and design** | **Data collection methods** | **Number and type of participants** |
| --- | --- | --- | --- | --- | --- |
| **Yarinbab et al., 2024 [102]** | Ethiopia, community and health facility | Explore male partners perspectives of maternity waiting homes, including their experiences of paternal support in rural Ethiopia. | Qualitative, Case study | Focus groups discussions, Interviews | 47 male partners (age 20 years or older) of women who had given birth 12 months prior to, and who lived with their spouses at the time of the study. |
| **Makusha, 2024 [16]** | South Africa, community | Explore the intricate interplay between young fatherhood, masculinities, and structural factors in South Africa. | Qualitative, Case study | Focus groups discussions, Interviews | 24 young fathers (age 18 to 24 years) |
| **Hampanda et al., 2024 [67]** | Kenya and Zambia, community and health facility | (1) Explore how expectant and recent fathers in couples affected by HIV from two settings of eastern and southern Africa perceive their involvement in family health with an emphasis on what is feasible and desirable given the cultural context and real-world life circumstances; (2) Identify the subjective factors reported by expectant/recent Kenyan and Zambian fathers that could be leveraged to motivate greater involvement in the promotion of family health. | Qualitative, Case study | Interviews | - 17 male partners (age 25 to 52 years) of postpartum women living with HIV (Kenya sample)  - 18 male partners (age 29 to 61 years) of pregnant women living with HIV (Zambia sample) |
| **Morgan et al., 2022 [61]** | Ghana, health facility | Explore the facilitators and barriers to husbands’ involvement in antenatal-related care in the Bosomtwe District of Ghana. | Qualitative, Case study | Interviews | - 14 husbands  - 14 pregnant women with or without birthing experience, and nursing mothers  - 6 biomedical midwives  - 2 TBAs  - All participants aged 25 to 55 years |
| **Mooij et al., 2022 [79]** | Tanzania, health facility | Explore the role of male partners in supporting women at the time of SAMM, and in the years after. | Qualitative, Cross-sectional study | Interviews, Hospital records | - 9 male partners (age 22 to 42 years) of women who experienced (pre-)eclampsia six to seven years before the study |
| **Boniphace et al., 2022 [117]** | Tanzania, community | Examine stories of shame, stigma or fear, which affects men’s ANC attendance together with their partners. | Qualitative, Case study | Focus groups discussions, Interviews | - 15 men with first-time pregnant wives  - 23 fathers with at least one child  - 1 biomedical health professional  - 2 community health workers  - 3 village leaders  - All participants aged 25 to 60 years |
| **Kaba et al., 2022 [62]** | Ethiopia, community and health facility | Explore the role of men in preventing perinatal HIV transmission in the Gambella region of Ethiopia. | Qualitative, Case study | Focus groups discussions, Interviews, Key informant interviews | - 63 husbands of pregnant women or recent mothers  - 10 pregnant women or recent mothers  - 9 stakeholders (opinion leaders, biomedical health professionals)  - Participants’ ages not specified |
| **Ampim et al., 2022 [63]** | Ghana, community and health facility | Examine how gender is done in the household when couples are expecting a baby and identify the extent to which acts of gender transgression may potentially take place during this peak in the reproductive lives of Ghanaian couples. | Qualitative, Case study | Focus groups discussions, Interviews | - 23 first-time expectant fathers (age 21 to 48 years)  - 13 pregnant women and 23 mothers (age 18 to 48 years) |
| **Mudi et al., 2021 [97]** | Nigeria, community | Explore the impact of the home visits on gender roles and decision-making and to evaluate to what extent the intervention was gender-transformative. | Qualitative, Case study | Narrative technique | - 23 women visited when they were pregnant (mean age 32 years)  - 21 men visited when their wives were pregnant (mean age 40 years) |
| **Lusambili, Wisofschi, et al., 2021 [105]** | Kenya, community | Explore how male engagement strategies influenced access to and utilization of RMNCH services. | Qualitative, Endline project evaluation study | Focus groups discussions, Interviews | - Specific sample numbers not provided except that 10 focus groups discussions were conducted separately with female and male community health volunteers, male and female adult community members, and female and male adolescent community members, and 11 interviews were conducted with male and female key informants at the county, sub-county and health facility levels.  - Participants’ ages not specified |
| **Lusambili, Muriuki, et al., 2021 [34]** | Kenya, community and health facility | Investigate the facilitators and barriers to working with men to promote RMNCH in partnership with women. | Qualitative, Case study | Focus groups discussions, Interviews | - As above |
| **Jeong et al., 2021 [115]** | Mozambique, community and health facility | Explore community perspectives regarding fathers’ roles in early child health services during the first three years of life to identify the barriers and facilitators to father involvement in Monapo District in northern, rural Mozambique. | Qualitative, Case study | Interviews, Observations | - 32 mothers and 4 fathers (ages 18 to 34 years)  - 15 biomedical health professionals  - 22 government and no-governmental organization service providers and managers |
| **Gibore & Gesase, 2021 [111]** | Tanzania, community | Examine views and knowledge on, and challenges to, men’s involvement in ANC services in the Dodoma Region of Tanzania. | Quantitative, Cross-sectional study | Survey questionnaire | - 966 men (ages 18 years or older) with a child below two years |
| **Chahalis et al., 2021 [119]** | Tanzania, community | Describe factors associated with men’s involvement in household tasks and explore the relationship between men’s help with tasks and women’s ANC-seeking, diet and workload during pregnancy as well as other health behaviors. | Quantitative, Cross-sectional study | Survey questionnaire | - 4217 men with a child below two years  - 4091 women with a child below two years  - All participant ages 18 years or older |
| **Boniphace et al., 2021 [112]** | Tanzania, community and health facility | Examine men’s perspectives on attending ANC with their pregnant partners in Misungwi District, Tanzania. | Qualitative, Case study | Focus groups discussions, Interviews | - 15 men with first-time pregnant wives and 23 fathers with at least one child (ages 25 to 60)  - 1 biomedical health professional  - 2 community health workers  - 3 village leaders |
| **Tinuola et al., 2020 [108]** | Nigeria, community and health facility | Explore perception of challenges that fathers face in their involvement in maternity care. | Mixed methods, Case study and Cross-sectional study | Focus groups discussions, Survey questionnaire | - 176 fathers and 146 mothers (age 18 to 64 years)  - 40 biomedical midwives |
| **Onyeze-Joe & Godin, 2020 [74]** | Nigeria, community and health facility | Understand the needs, experiences and perceptions of pregnancy and childbirth by first-time fathers and the impact of their perceptions and needs on their actual involvement in pregnancy and delivery in Nigeria. | Qualitative, Case study | Interviews | - 50 men (age 23 to 38 years) |
| **McLean, 2020 [31]** | Sierra Leone, community and health facility | Highlight men’s subjective experiences of pregnancy and childbirth, with attention to the ways in which men strive to enact both effective and socially meaningful forms of partner support. | Qualitative, Ethnographic study | Interviews, Life histories, Observations | - 106 fathers (age 18 to 39) |
| **Maluka, Joseph, et al., 2020 [129]** | Tanzania, community | Understand the factors that lead to delay in seeking ANC services among pregnant women in Tanzania. | Qualitative, Case study | Focus groups discussions, Interviews | - Men and women (age 15 to 49 years) who participated in 40 focus groups: specific numbers not provided.  - 80 biomedical health professionals interviewed. |
| **Maluka, Japhet, et al., 2020 [98]** | Tanzania, community | Report participatory action research aimed to promote male participation in pregnancy and childbirth in Iringa Region, Tanzania. | Qualitative, Case study | Community meetings, Interviews, Survey questionnaire | - 400 women (aged 15 or older) with experience in using MCH services, were pregnant or had given birth in the last 12 months  - 200 men (age not specified)  - 40 community health workers  - 450 stakeholders (biomedical health professionals, non-health professionals, community leaders, representatives of men’s, women’s and youth groups, etc.) |
| **Mabachi et al., 2020 [80]** | Kenya, health facility | Understand the types of social support Kenyan men reported providing their HIV-positive partners. | Qualitative, Case study | Focus groups discussions | - 33 male partners of pregnant women with HIV (age not specified) |
| **Gibore & Bali, 2020 [99]** | Tanzania, community | Explore community perspectives on potential barriers to men’s involvement in maternity care in central Tanzania. | Qualitative, Case study | Focus groups discussions, Interviews | - 236 couples (the woman must have had at least two pregnancies at the time of the study)  - 20 community leaders  - 11 biomedical health professionals  - 3 village health workers  - Participants’ ages not specified |
| **Comrie-Thomson et al., 2020 [118]** | Tanzania and Zimbabwe, community | Describe how male involvement interventions can foster more loving couple relationships and explore how these changes in couples’ emotional relationships may be a plausible mechanism supporting the overall effectiveness of male involvement interventions. | Qualitative, Case study | Focus groups discussions, Interviews | - 47 adult men (age 24 to 76)  - 34 adolescent boys (age 15 to 19)  - 46 adult women (age 20 to 57)  - 44 adolescent girls (age 15 to 19) |
| **Al-Mujtaba et al., 2020 [96]** | Nigeria, community and health facility | Present, from the male perspective, factors influencing women’s access to maternal-child health and PMTCT services in North-Central Nigeria. | Qualitative, Case study | Focus groups discussions | - 30 married or partnered men (age 18 years or older) |
| **Saah et al., 2019 [38]** | Ghana, community and health facility | Explore male partner involvement in skilled birth at the North Dayi District of Ghana. | Qualitative, Case study | Interviews | - 14 couples (women attending the targeted child welfare clinic and their male spouses) (age 24 to 46 years)  - 2 biomedical health professionals |
| **Ongolly & Bukachi, 2019 [109]** | Kenya, community and health facility | Explore the barriers to men’s involvement in ANC and PNC in Butula sub-county, western Kenya. | Mixed methods, Cross-sectional study | Focus groups discussions, Survey questionnaire | - 96 men (age 20 or 83)  - 4 biomedical health professionals |
| **Muheirwe & Nuhu, 2019a [113]** | Uganda, health facility | Understand the health facilities infrastructures and programs encouraging or discouraging men's participation in MCH. | Mixed methods, Case study | Document analysis, Focus groups discussions, Interviews, Observations, Survey questionnaire | - 77 fathers and male partners (age 18 to 51+ years)  - 83 mothers and female spouses (age 18 to 51+ years)  - 8 key informants (two TBAs, two Village Health Team members, four biomedical health professionals) |
| **Muheirwe & Nuhu, 2019b [123]** | Uganda, community and health facility | Explore the community perspectives towards men’s participation in MCH in Kabale district, Western Uganda | Mixed methods, Case study | Document review, Focus groups discussions, Interviews, Observations, Survey questionnaire | - As above |
| **Kayongo & Miller, 2019 [122]** | Uganda, community | Investigate men’s response to campaign messaging in this campaign (*Obulamu: How’s Your Pregnancy?*), and factors associated with that response. | Qualitative, Case study | Focus groups discussions, Interviews | - 64 men (age 18 to 50 years) who had babies below one year or expectant wives, and pregnant women and mothers with babies below one year: the numbers of each participant group were not provided. |
| **Greenspan et al., 2019 [81]** | Tanzania, community and health facility | Draw on interviews with men in Morogoro Region, Tanzania to describe their role in maternal and newborn health care seeking. | Qualitative, Case study | Interviews | - 27 male partners (age 22 to 60 years) of recent mothers |
| **Galle et al., 2019 [73]** | Mozambique, community and health facility | Explore the attitudes, practices and beliefs of health policymakers, health care providers and local communities regarding the benefits, challenges, risks and approaches to increase men’s involvement during pregnancy in southern Mozambique. | Qualitative, Case study | Focus groups discussions, Interviews | - 10 expectant and recent fathers and mothers  - 22 expectant women and recent mothers  - 7 traditional care providers (one traditional healer, six TBAs)  - 17 community leaders  - 7 community health workers and local activists  - 46 health professionals  - Participants’ ages not specified |
| **Cheptum et al., 2019 [69]** | Kenya, health facility | Analyze women’s perception of men’s role in birth preparedness in Migori County, Kenya. | Qualitative, Case study | Focus groups discussions | - 32 pregnant women (age 16 to 33 years) |
| **Wombeogo & Ayembilla, 2018 [90]** | Ghana, community | Ensure that husbands take active role in assisting their pregnant wives obtain maximum support to reduce maternal, neonatal and child health risk in Ghana. | Mixed methods, Cross-sectional study | Focus groups discussions, Interviews, Survey questionnaire | - 199 husbands (age 18 to 50+ years) with pregnant wives of wives who had at least one child below age one  - 199 wives (ages not specified clearly) who were pregnant or had at least one child below age one |
| **Treacy et al., 2018 [77]** | Sierra Leone, community and health facility | Explore the perceptions and decision-making processes of women and their communities during childbirth in rural Sierra Leone. | Qualitative, Case study | Focus groups discussions, Interviews | - 42 men and women who participated in focus groups: number not disaggregated by sex.  - 22 key informants interviewed: ten mothers, biomedical health professionals, one village chief, 3 TBAs, and a group of motorbike drivers)  - Participants’ ages not specified |
| **Musoke et al., 2018 [93]** | Kenya, health facility | Explore men’s views on the time surrounding pregnancy and birth, and male involvement in antenatal care and prevention of mother-to-child transmission services; explore facilitators and barriers to men’s involvement in rural Kenya, including their personal hopes, fears and challenges. | Qualitative, Intervention pilot study | Interviews | - 40 male partners (age 18 to 70 years) of HIV positive and HIV negative pregnant women |
| **Maluka & Peneza, 2018 [82]** | Tanzania, health facility | Describe perceptions about male involvement in pregnancy and childbirth in Masasi District in Tanzania. | Qualitative, Case study | Interviews | - 13 male partners  - 20 pregnant women and recent mothers  - 7 biomedical health professionals  - 10 village and religious leaders  - 3 TBAs  - Participants’ ages not specified |
| **Aborigo et al., 2018 [64]** | Ghana, community and health facility | Explore the reasons for men’s resistance to the adoption of a more proactive role in pregnancy care and their enduring influence in the decision-making process during emergencies. | Qualitative, Case study | Focus groups discussions, Interviews | - 16 biomedical health professionals  - 120 community opinion leaders  - Participants’ ages not specified |
| **Aarnio et al., 2018 [71]** | Malawi, community | Provide information about husbands’ role in decision-making and healthcare seeking in cases of pregnancy complications in Mangochi district, Malawi. | Qualitative, Cross-sectional study | Interviews | - 12 husbands and wives of married couples (ages not specified)  - 6 key informants: two TBAs, one village chief and his wife, and the  mother and uncle (head of clan) of one participant (wife) |
| **Sileo et al., 2017 [86]** | Uganda, health facility | Explore factors influencing male support for and participation in women’s reproductive health services and elicit suggestions for strategies to increase male participation. | Qualitative, Case study | Focus groups discussions | - 36 men and 40 women (average age 29 years) |
| **Morgan et al., 2017 [85]** | Uganda, community | Explore the root causes of women’s lack of maternal health care access and utilization. | Qualitative, Cross-sectional study | Focus groups discussions | - 7 younger mothers (age 15 to 25 years)  - 9 older mothers (age 26 to 55 years)  - 8 fathers (age not specified)  - Transporters (age not specified) |
| **Matseke et al., 2017 [78]** | South Africa, health facility | Explore the meaning and understanding of MPI among South African men and to consider strategies for culturally appropriate integration of male partners in antenatal and postnatal care programs in rural clinics in Mpumalanga. | Qualitative, Grounded theory | Focus groups discussions | - 53 men (age 26 to 50 years) |
| **Manda-Taylor et al., 2017 [72]** | Malawi, community | Explore the role and involvement of men in MNCH services, as part of the broader understanding of those community system factors. | Mixed methods, Case study | Focus groups discussions, Interviews | - 5 husbands  - 12 pregnant women and recent mothers  - 3 grandmothers and mothers-in-law  - 48 biomedical health professionals, government officials, and non-governmental organization officials)  - 31 traditional and religious leaders  - 6 TBAs  - Participants’ ages not specified |
| **Lowe, 2017 [89]** | The Gambia, community | Explore some of the underlying social and cultural factors affecting husbands’ involvement in maternal health issues pertaining to pregnancy and delivery in rural Gambia. | Qualitative, Case study | Focus groups discussions, Interviews | - 50 married men (ages not specified))  - 6 TBAs (age not specified) |
| **Flax et al., 2017 [94]** | Malawi and Uganda, health facility | Examine the interplay of gender and individual, inter-personal, health system, and community factors that contribute to PMTCT participation in Malawi and Uganda. | Qualitative, Case study | Focus groups discussions, Interviews | - 64 PMTCT women (32 in Malawi and Uganda apiece)  - 48 lost to follow up women (32 in Malawi, 16 in Uganda)  - All participants aged 18 years or older |
| **Bougangue & Ling, 2017 [65]** | Ghana, community | Provide opportunity for men to share their views about involvement in various aspects of maternal health care, ranging across pregnancy, delivery and postnatal care. | Qualitative, Thematic analysis | Focus groups discussions, Interviews | - 93 husbands (including 5 traditional leaders and 10 community health volunteers)  - 5 biomedical community health officers  - Participants’ ages not specified |
| **Vermeulen et al., 2016 [83]** | Tanzania, health facility | Gain an understanding of male perceptions, attitudes and behaviour regarding their involvement in pregnancy and ANC, prior to implementation of the WCCP in a rural district in Tanzania. | Mixed methods, Case study | Focus groups discussions, Interviews, Survey questionnaire | - 64 husbands (age 18 to 64 years)  - 181 pregnant women (age 18 to 54 years)  - 6 biomedical health professionals  - 7 TBAs |
| **Turinawe et al., 2016 [101]** | Uganda, community | Explore the interaction between men and TBAs in shaping maternal healthcare in a rural Ugandan context. | Qualitative, Ethnographic study | Focus groups discussions, Interviews, Observations | - Women, men and male village health team members who participated in 12 focus groups: specific number for each group not provided  - 12 interviewees: five TBAs, three village health team members, two men, and two biomedical health professionals.  - Participants’ ages not specified |
| **Nyandieka et al., 2016 [68]** | Kenya, health facility | Report part findings of a study conducted to assess health priority setting process and its implication on availability, access, and use of emergency obstetric care ervices in Malindi. | Qualitative, Case study | Focus groups discussions, Interviews | - Community members who participated in 7 focus groups: specific numbers not provided.  - 22 interviews with biomedical health professionals and managers, women seeking services at the facilities, TBAs, CHWs, and opinion  leaders.  - Participants’ ages not specified |
| **Lowe et al., 2016 [103]** | The Gambia, community | Depict the gender dynamics in a rural Gambian context by exploring the social and cultural factors affecting maternal health. | Qualitative, Case study | Focus groups discussions, Interviews, Observations | - 50 mothers (age 15 to 30 years) with at least one child.  - 6 TBAs (ages not specified) |
| **Ganle et al., 2016 [120]** | Ghana, community | Examine women's perspectives on men's involvement in maternal and child healthcare in Ghana, focusing on the Upper West Region as a case example. | Qualitative, Case study | Focus groups discussions | - 125 pregnant women and lactating mothers (age 18 to 49 years) |
| **Brubaker et al., 2016 [92]** | Kenya, community and health facility | Better understand the beliefs of men and women in western Kenya regarding the appropriate role of men in maternal health and to identify barriers to greater involvement. | Qualitative, Cross-sectional study | Focus groups discussions | - 40 expectant fathers and fathers (age 18 to 50 years) with at least one child  - 70 mothers (age 15 to 40 years) with at least one child  - 24 community health workers (age not specified) |
| **Audet et al., 2016 [116]** | Mozambique, community and health facility | Explore barriers and facilitators of ANC uptake and HIV counseling and testing during pregnancy as well as generate guidance for the development of culturally situated intervention strategies to improve service uptake | Qualitative, Case study | Focus groups discussions | - 99 community members (50 men [median age 46.5 years], 49 women [median age 37 years])  - 23 biomedical health professionals (age not specified) |
| **Ganle & Dery, 2015 [114]** | Ghana, community | Explore the barriers to, and opportunities for, men’s involvement in maternal healthcare in Ghana, focusing on the Upper West Region. | Qualitative, Case study | Focus groups discussions, Interviews | - Adult men: total number not specified except that these men participated in 12 focus groups, each with 7–12 participants.  - 30 key informants (six chiefs, five women leaders, six assemblymen, five community health nurses, six community health officers, two mother-to-mother support group leaders)  - All participants age from 20 to 50 years |
| **Ganle et al., 2015 [128]** | Ghana, community and health facility | Examine how intra-familial decision-making affects women’s ability to access and use maternal health services. | Qualitative, Case study | Focus groups discussions, Interviews | - 185 expectant and lactating mothers (age 18 to 45 years)  - 20 biomedical health professionals (ages not specified) |
| **Azuh et al., 2015 [95]** | Nigeria, community | Understand the influence of socio-cultural factors influencing health care utilization among women during pregnancy and childbirth. | Quantitative, Cross-sectional study | Survey questionnaire | - 260 married women (age 15 to 49 years) with at least one live birth two years prior to the study |
| **Singh et al., 2014 [87]** | Uganda, community and health facility | Explore the barriers, and spaces for, male involvement in Uganda in maternal healthcare; examine the current participation of men during pregnancy and delivery; describe women’s attitudes towards male involvement during pregnancy and delivery; and explore the opportunity for openness, dialogue, more responsibility sharing during pregnancy and delivery. | Mixed methods, Cross-sectional study | Focus groups discussions, Survey questionnaire | - 12 expectant and recent fathers  - 23 expectant women and recent mothers  - All participants aged 18 years or older |
| **Moyer et al., 2014 [91]** | Ghana, community and health facility | Explore the impact of social factors on place of delivery in northern Ghana, with specific focus on the impact of community and familial social structures and the role of traditional cultural practices surrounding childbirth. | Qualitative, Case study | Focus groups discussions, Interviews | - 35 women with a newborn infant  - 13 biomedical health professionals  - 8 traditional care providers (4 TBAs, 4 herbalists)  - 16 community leaders  - 103 extended family members (81 grandmothers, 22 compound heads)  - Participants’ ages not specified |
| **McMahon et al., 2014 [84]** | Tanzania, community | Explore how rural Tanzanian women, and their male partners describe disrespect and abuse experienced during childbirth in facilities and how they respond to abuse in the short or long-term. | Qualitative, Cross-sectional study and Grounded theory | Interviews | - 49 women who delivered a child within 14 months prior to the study  - 27 male partners  - 20 community health workers  - 5 community leaders  - 11 religious leaders  - Participants’ ages not specified |
| **Dumbaugh et al., 2014 [66]** | Ghana, community | Increasing knowledge and under- standing of the potential for male involvement in newborn care in a rural African setting. | Qualitative, Case study | Focus groups discussions, Interviews | - 34 (recent) fathers (age 20 to 80 years)  - 25 recent mothers (age 15 to 40 years) |
| **Doyle et al., 2014 [76]** | Rwanda, community | Highlight authors’ experiences of implementing the MenCare+ programme in Rwanda, a collaboration between Promundo and the Rwanda Men’s Resource Center. | Qualitative, Case study | Focus groups discussions, Fathers’ education groups, Public testimonies | - 600 men and fathers (age 15 to 35 years) |
| **Somé et al., 2013 [124]** | Burkina Faso, community | Examine how decisions for maternal care are made in two rural communities in Burkina Faso. | Qualitative, Case study | Focus groups discussions, Interviews | - 30 women (age 15 to 49 years) who used or did not use a health facility for maternal care and were from the ethnic groups of Yana for Ouargaye and Gourmanthé for Diapaga. |
| **Kwambai et al., 2013 [70]** | Kenya, community | Provide insight into men’s perceptions of maternal health care services and identify factors that facilitate or constrain men’s involvement in ANC and delivery care in western Kenya. | Qualitative, Case study | Focus groups discussions | - 68 married men (age 20 to 65) |
| **Gross et al., 2013 [127]** | Tanzania, community | Investigate men and women’s discussions about men’s roles and responsibilities in prenatal care and link them to a literature-based analysis of changing norms and values at the household level and beyond. | Qualitative, Case study | Focus groups discussions | - 85 men and women: specific numbers and ages for each gender not provided. |
| **Aarnio et al., 2013 [39]** | Malawi, community | Explore how husbands perceive delivery care in rural Malawi. | Mixed methods, Cross-sectional study | Survey questionnaire | - 389 ever-married men whose wives (age 15 to 49 years) and had been pregnant in the 5 years prior to the study. |
| **Kululanga et al., 2012 [107]** | Malawi, community and health facility | Examine the core causes of barriers to husbands’ involvement in maternal health care in rural Malawian settings. | Qualitative, Case study | Focus groups discussions, Interviews | - 40 men (age 22 to 55 years)  - 60 women (age 18 to 39 years)  - 2 biomedical health professionals  - 6 key informants (village headman, TBAs, village health committee members, traditional counsellors, elderly men and women) |
| **Abass et al., 2012 [104]** | Ghana, community | Assess the effects of socio-cultural practices on male involvement in reducing maternal mortality in Savelugu-Nanton District (Ghana). | Mixed methods, Case study and Cross-sectional study | Focus groups discussions, Interviews, Observations | - 210 household heads (107 females, 103 males)  - Key informants were interviewed (biomedical health professionals, TBAs, traditional leaders): specific number not specified.  - Participants’ ages not specified |
| **Falnes et al., 2011 [100]** | Tanzania, community and health facility | Explore the acceptability of the PMTCT programme components and identify structural and cultural challenges to male involvement. | Mixed methods, Cross-sectional study | Focus groups discussions, Interviews, Survey questionnaire | - 446 mothers (survey)  - Mothers and fathers who participated in nine focus groups of 5–12 pax per group: specific total numbers not provided.  - 21 interviewees (5 fathers, 5 mothers, 11 biomedical health professionals)  - All participants aged 18 years or older |
| **Adeleye et al., 2011 [75]** | Nigeria, community | Describe the development and implementation process of the Ekialodor safe motherhood program and analyze how group health talks used local culture and gender norms to engage male leaders in trying to improve maternal health in the community. | Qualitative, Cross-sectional study | Small-group health talks | - 190 elders (average age 60 years) and young adult men (average age 30 years)  - Specific numbers not disaggregated by participant group |
| **Reece et al., 2010 [106]** | Kenya, health facility | Assess men’s and women’s perceptions of the factors that influence the likelihood that male spouses will support their wives during pregnancy, and themselves become engaged with PMTCT-related antenatal care initiatives. | Qualitative, Cross-sectional study | Focus groups discussions | - 31 male spouses of PMTCT enrollees  - 44 HIV-positive men from support groups  - 37 HIV-positive women enrolled in PMTCT  - 34 HIV-negative women enrolled in PMTCT  - Participants’ ages not specified |
| **Byamugisha et al., 2010 [88]** | Uganda, community | Determine the level of participation of male partners in the PMTCT programme and to identify factors that determine male participation in this programme. | Mixed methods, Cross-sectional study | Focus groups discussions, Interviews, Survey questionnaire | - 388 men (age 18 years or older) |
| **Theuring et al., 2009 [33]** | Tanzania, community and health facility | Learn about men’s perspectives on and experiences with ANC and PMTCT services in Mbeya Region, Tanzania, in order to identify strategies for increasing partner participation rates in those services. | Mixed methods, Case study and Cross-sectional study | Focus groups discussions, Interviews, Survey questionnaire | - 124 men interviewed (age 22 to 59 years)  - Men and women who participated in focus groups: specific numbers not specified. |
| **Olayemi et al., 2009 [121]** | Nigeria, health facility | Assess the level of pregnancy and birth participation in Nigerian men, the possible associations, the attitude of the women and likely targets for improved care delivery. | Quantitative, Cross-sectional study | Survey questionnaire | - 462 pregnant women (mean age 30.5 years) |
| **Pembe et al., 2008 [126]** | Tanzania, community and health facility | Describe perceptions of maternal referrals within the community and among health staff in the rural district of Rufiji in Tanzania. | Qualitative, Case study | Focus groups discussions | - 85 male and female community members who participated in 8 focus groups: sample not disaggregated by sex.  - 11 biomedical health professionals  - Participants’ ages not specified |
| **Mbweza et al., 2008 [41]** | Malawi, community | Examine the decision-making processes of husband-and-wife dyads in matrilineal and patrilineal marriage traditions of Malawi in the areas of money, food, pregnancy, contraception, and sexual relations. | Qualitative, Grounded theory | Interviews, Survey questionnaire | - 60 husbands and wives (30 couples) aged 20 to 53 years |
| **Jansen, 2006 [125]** | Ghana, community | Describe and understand the traditional structures of childbirth in Kwame Danso/Ghana and explore why the pregnant women do not make use of supervised deliveries in the modern institutions. | Qualitative, Mini-ethnographic study | Interviews, Observations | - 5 TBAs (ages 43 to 76 years) |
| **Odimegwu et al., 2005 [40]** | Nigeria, community | Determine the level of awareness and involvement of male spouses in emergency obstetric conditions of their partners. | Quantitative, Cross-sectional study | Focus groups discussions, Interviews, Survey questionnaire | - 1720 husbands (mean age 37 years)  - 1920 wives (mean age 29.1 years) |
| **Mullick et al., 2005 [110]** | South Africa, health facility | Discuss provider and client views or perspectives about male involvement during maternity, providers’ motivational factors, lessons learnt, opportunities identified, and recommendations and implications. | Quantitative, Intervention study | Focus groups discussions, Survey questionnaire | - 2082 women (1087 control group, mean age 23 years; 995 intervention group, mean age 24 years)  - 584 male partners  of the women in the intervention group (mean age 28 years)  - Biomedical health professionals: number and ages not specified. |

*Note*: * = number in brackets in front of each article corresponds to its number in the references list; ANC = antenatal care; HIV = human immune-deficiency virus; MCH = maternal and child health; MNCH = maternal, neonatal, and child health; MPI = male partner involvement; PMTCT = prevention of mother-to-child transmission of HIV; PNC = postnatal care; RMNCH = reproductive, maternal, neonatal, and child health; SAMM = severe acute maternal morbidity; TBA = traditional birth attendant; WCCP = Woman Centered Care Project
